# Droplet Impact-Based Microliter Viscometry

**DOI:** 10.1021/acs.analchem.5c00471

**Published:** 2025-06-16

**Authors:** Shuxian Tang, Xiang Li, Wanying Wang, Wenchang Zhao, Ying Zhou, Shiyu Wang, Yanhong Li, Peng Yu, Xiewen Wen, Guohui Hu, Pingan Zhu

**Affiliations:** † Department of Mechanical Engineering, 53025 City University of Hong Kong, 999077 Hong Kong, China; ‡ Department of Mechanics and Aerospace Engineering, 255310 Southern University of Science and Technology, 518055 Shenzhen, China; § Department of Biomedical Sciences, City University of Hong Kong, 999077 Hong Kong, China; ⊥ State Key Laboratory of Ultra-precision Machining Technology, Department of Industrial and Systems Engineering, 26680 The Hong Kong Polytechnic University, 999077 Hong Kong, China; ∥ Shanghai Institute of Applied Mathematics and Mechanics, School of Mechanics and Engineering Science, Shanghai Key Laboratory of Mechanics in Energy Engineering, Shanghai Frontier Science Center of Mechanoinformatics, 34747 Shanghai University, 200072 Shanghai, China

## Abstract

Characterizing liquid viscosity and rheological properties with small sample volumes is crucial in fields where liquid samples are often limited, such as biological fluids for biomedical diagnostics and trace chemical products. However, traditional viscometers often require large sample volumes, and many existing small-volume viscometry techniques fall short in analyzing non-Newtonian fluids due to their limited shear rate range. While microfluidics-based viscometers offer flexibility in shear rate control, they are generally associated with intricate fabrication processes and high costs. Here, we introduce a droplet impact-based microliter viscometry (DI-μV), building on a quantitative relationship between viscosity and the maximum spreading factor of droplets impacting super-repellent surfaces. Leveraging the sufficient viscous effect during droplet impact, DI-μV can measure effective viscosity across varying effective shear rates and probe the rheological properties of liquids, using only μL-scale volumes. As a tool for viscosity measurement, DI-μV exhibits minimal sample consumption, adjustable effective shear rate, self-cleaning capabilities, operational simplicity, and cost-effectiveness, thereby offering relevant practical implications.

## Graphical abstract

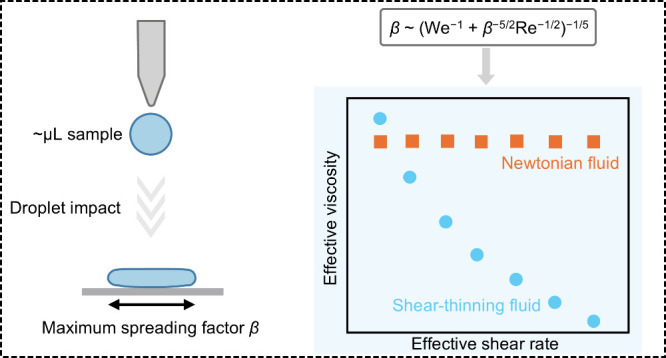

## Introduction

Viscosity is one of the most fundamental properties of liquids, as it governs their flow behavior. Measuring viscosity is essential across diverse domains, including product quality control in industries such as food^1^ and pharmaceuticals,^2^ biological fluid analysis for disease diagnostics,^3,4^ pipeline transportation,^5^ as well as fundamental research in rheology. Beyond viscosity itself, how viscosity varies with shear rate is another critical property, especially for non-Newtonian fluids, whose viscosity can be shear rate-dependent.^6^ Performing viscosity measurement over a range of shear rates is crucial to comprehensively characterize the rheological profile of various liquid samples.

However, traditional viscometers, such as rotational, capillary, and falling ball viscometers, require large sample volumes, often at the milliliter (mL) scale, which limits their utility for analyzing precious or scarce samples.^7,8^ Such samples may involve biological fluids, pharmaceuticals, and trace products from chemical syntheses.^9^ Recent advances in the development of small-volume viscometry have enabled measurements with sample volumes as small as approximately 100 μL or less. Nevertheless, these microviscometers exhibit certain deficiencies. For instance, the small-volume viscometry techniques based on vibration,^10^ diffusion,^11^ droplet sliding,^12^ and liquid film movement,^13^ have invariable or narrow shear rate range, making them unsuitable for characterizing the shear-dependent flow behavior of non-Newtonian fluids. Although microfluidics-based viscometers can achieve a wide shear rate range,^7^ they rely on precisely engineered micrometer-scale channels,^14,15^ and many require expensive and labor-intensive processes like soft lithography for the fabrication of microfluidic chips.^16–19^ Moreover, the difficulty in cleaning microchannels further exacerbates costs, especially in single-use applications, such as those involving infectious biological samples.^3^ Therefore, there is a pressing need for developing techniques that are versatile in shear rate variation, user-friendly, and cost-effective, for analyzing viscosity properties in a μL-volume way.

The impact of droplets on solid surfaces is a well-studied phenomenon.^20–25^ When a droplet falls vertically onto a solid surface, it spreads horizontally upon contact, generating a strain that evolves throughout the spreading process.^26^ The strain rate is influenced by the droplet’s impact velocity, which can be easily adjusted, for instance, by varying the droplet’s falling height. The droplet continues to spread until it reaches the maximum spreading, a key parameter extensively investigated for its critical role in applications like inkjet printing. Numerous endeavors have been devoted to establishing models for predicting the maximum spreading factor β, defined as the ratio between the maximum diameter *D*_m_ and the initial diameter *D*_0_, β = *D*_m_/*D*_0_.^27–29^ Although many studies have explored the impact of droplets with varying viscosities and confirmed that viscosity significantly affects the maximum spreading, existing models fail to accurately predict β across a wide viscosity range.^30–36^ Consequently, a reliable quantitative framework that links viscosity to β is yet to be developed. Given that a single droplet typically has a small volume—approximately 4 μL for a droplet with a diameter of 2 mm—the observable influence of viscosity on droplet spreading opens up new possibilities. Specifically, if a quantitative relationship between viscosity and β can be established, droplet impact could become the foundation for a microviscometer with an adjustable shear rate.

Herein, we introduce an approach for characterizing liquid viscosity by leveraging droplet impact dynamics, focusing specifically on the relationship between viscosity and β. Several Newtonian and shear-thinning (non-Newtonian) fluids with different viscosity values and rheological properties were prepared, with their shear viscosity determined using a conventional rotational rheometer for reference. Droplet impact experiments on a super-repellent surface were conducted to measure β for these liquids under varying impact velocities. Initially, the experimental results for β were examined using several predictive models commonly cited in the literature. Subsequently, we developed a semiempirical model through scaling analysis to rationalize our experimental data. From our model, a quantitative correlation between viscosity and β was established, and the error sources of viscosity calculation were systematically analyzed. Furthermore, we proposed a droplet impact-based microliter viscometry (DI-μV) and evaluated its capability to measure effective viscosity values at different effective shear rates in a μL-volume way.

## Experimental Section

### Preparation of Liquid Samples

Distilled water and glycerol–water mixtures were used as the Newtonian fluids in this study. To achieve varying viscosity levels, the concentration of glycerol (≥99.5%, Aladdin) was adjusted for glycerol–water mixtures. The mixtures were labeled Gly-50, Gly-80, and Gly-90, according to their glycerol concentration (Table 1). Aqueous polymer solutions of carboxymethyl cellulose (CMC, Mw ∼ 700 kDa, Aladdin) were used as the shear-thinning (non-Newtonian) fluids. Two different concentrations of CMC solution were prepared, denoted as CMC-0.04 and CMC-0.2, based on their respective concentrations (Table 1).

**Table 1:** Composition and Physical Properties of Liquid Samples

Liquid	Composition	η (mPa·s)	ρ (g cm^–3^)	σ (mN m^–1^)
Water	Pure distilled water	0.89	0.997	72.702
Gly-50	Glycerol–water mixture (50 wt.% glycerol)	5.127	1.124	66.252
Gly-80	Glycerol–water mixture (80 wt.% glycerol)	44.81	1.206	64.006
Gly-90	Glycerol–water mixture (90 wt.% glycerol)	154.2	1.230	62.904
CMC-0.04	Aqueous solution of CMC (0.04 wt.% CMC)	Shear-dependent	0.997	70.394
CMC-0.2	Aqueous solution of CMC (0.2 wt.% CMC)	Shear-dependent	0.997	71.019

### Measurements of Shear Viscosity

The viscosity properties of the prepared liquid samples were examined using a rotational rheometer (Kinexus pro+, Malvern, UK), equipped with a cone-plate geometry with a diameter of 50 mm and an angle of 1°. Shear viscosity measurements were performed in the steady shear flow mode, covering a shear rate range of 100 to 1000 s^–1^ for glycerol–water mixtures and 3.98 to 3981 s^–1^ for CMC solutions. All measurements were conducted at a controlled temperature of 25 °C. For CMC solutions, the shear viscosity data were fitted using the Carreau–Yasuda model:

$$\eta \left(\mathrm{\gamma ̇}\right)=\eta_{\infty }+\left(\eta_{0}-\eta_{\infty }\right){\left[1+{\left(\lambda \mathrm{\gamma ̇}\right)}^{b}\right]}^{\left(n-1\right)/b}$$

where η­(*γ̇*) is the shear viscosity, η_∞_ is the infinite-shear viscosity, η_0_ is the zero-shear viscosity, λ is the time constant, *n* is the power-law index, and *b* is the Yasuda parameter, respectively. The fitted parameters for the CMC-0.04 solution were η_∞_ = 2.37 mPa·s, η_0_ = 27.59 mPa·s, λ = 0.0058 s, *n* = 0, and *b* = 0.51. For CMC-0.2, the fitted parameters were η_∞_ = 3.29 mPa·s, η_0_ = 105.9 mPa·s, λ = 0.026 s, *n* = 0.36, and *b* = 0.67.

### Preparation of Super-Repellent Surfaces

Super-repellent surfaces were prepared by depositing fluorinated nanoparticles on glass. To prepare the fluorinated nanoparticle suspension, the following steps were performed: (i) 45 mL of ethanol (absolute; Anaqua) and 15 mL of aqueous ammonia (28–30%; Aladdin) were mixed, (ii) 4 mL of tetraethyl orthosilicate (TEOS, > 99%; Macklin) were added dropwise and stirred at 60 °C for 3 min, and (iii) 0.2 mL of 1H,1H,2H,2H-perfluorodecyltriethoxysilane (>98.0%; TCI) was added, and the mixture was stirred for 24 h. Subsequently, 1 mL of the prepared fluorinated nanoparticle suspension was added onto a glass slide (2.5 cm × 2.5 cm), which was then dried in an oven at 50 °C for 1 h to obtain the superamphiphobic surface.

### Droplet Impact

Each liquid sample was loaded into a syringe connected to a needle via a flexible tube. The needle was then mounted on an adjustable sliding rail to control the release height of droplets, meanwhile ensuring a perpendicular orientation to the horizontal plane. Needle gauges ranging from 30 to 34 G were selected based on the ease of droplet formation depending on the fluid resistance. A pump (LSP01–1A, Longer Pump) was used to drive the liquids. Following generation, the droplets fell vertically from the needle tips and impacted the super-repellent surface below. The impact events were captured using a high-speed camera (Fastcam Mini UX100, Photron) at 10,000 or 20,000 frames per second, depending on the video clarity. The impact dynamics of droplets were analyzed using ImageJ (National Institutes of Health) to determine parameters including the impact velocity (*U*), initial diameter (*D*_0_), and maximum spreading diameter (*D*_m_). The typical *D*_0_ of generated droplets is 2.11 mm, 2.11 mm, 2.04 mm, 2.55 mm, 2.18 mm, and 2.25 mm for Water, Gly-50, Gly-80, Gly-90, CMC-0.04 and CMC-0.2, respectively. The impact experiments were performed at room temperature.

### Measurement of Density and Surface Tension

To determine the density of the liquid samples, 5 mL volumetric flasks were initially filled to the calibration mark with distilled water, which has a known density of 0.997 g cm^–3^. The net weight of the water was recorded, and this value was divided by its density to ascertain the exact calibrated volume of the volumetric flasks. After thorough cleaning and drying, these flasks were used for the density measurement of the liquid samples. The net weight of a liquid sample required to fill in a flask to its calibration mark was measured, and this weight was divided by the corresponding calibrated volume of the flask to calculate the sample’s density. The surface tension of liquid samples was assessed by the pendant drop method using a goniometer (SDC-350, Sindin).

## Results and Discussion

### Viscosity Calibration by a Rheometer

To comprehensively examine the intrinsic relationship between droplet spreading dynamics and viscosity, we utilized liquid samples with different viscosity values and rheological properties, involving both Newtonian and shear-thinning (non-Newtonian) fluids. The Newtonian fluids were simulated using simple fluids including water and glycerol–water mixtures, and the shear-thinning fluids were represented by polymer solutions of CMC, as summarized in Table 1. For calibration purposes, the shear viscosity of these fluids was initially measured using a rotational rheometer.

Figure 1a presents the shear viscosity of glycerol–water mixtures with different glycerol concentrations across a shear rate range, all showing constant apparent viscosities that confirm their Newtonian behavior. As for water, one of the most common Newtonian fluids, its viscosity value was obtained from the literature.^36^ The viscosity of glycerol–water mixtures increases with the glycerol concentration. Accordingly, a series of Newtonian fluids with viscosity spanning from 0.89 to 154.2 mPa·s was prepared for subsequent droplet impact experiments.

**Figure 1:**
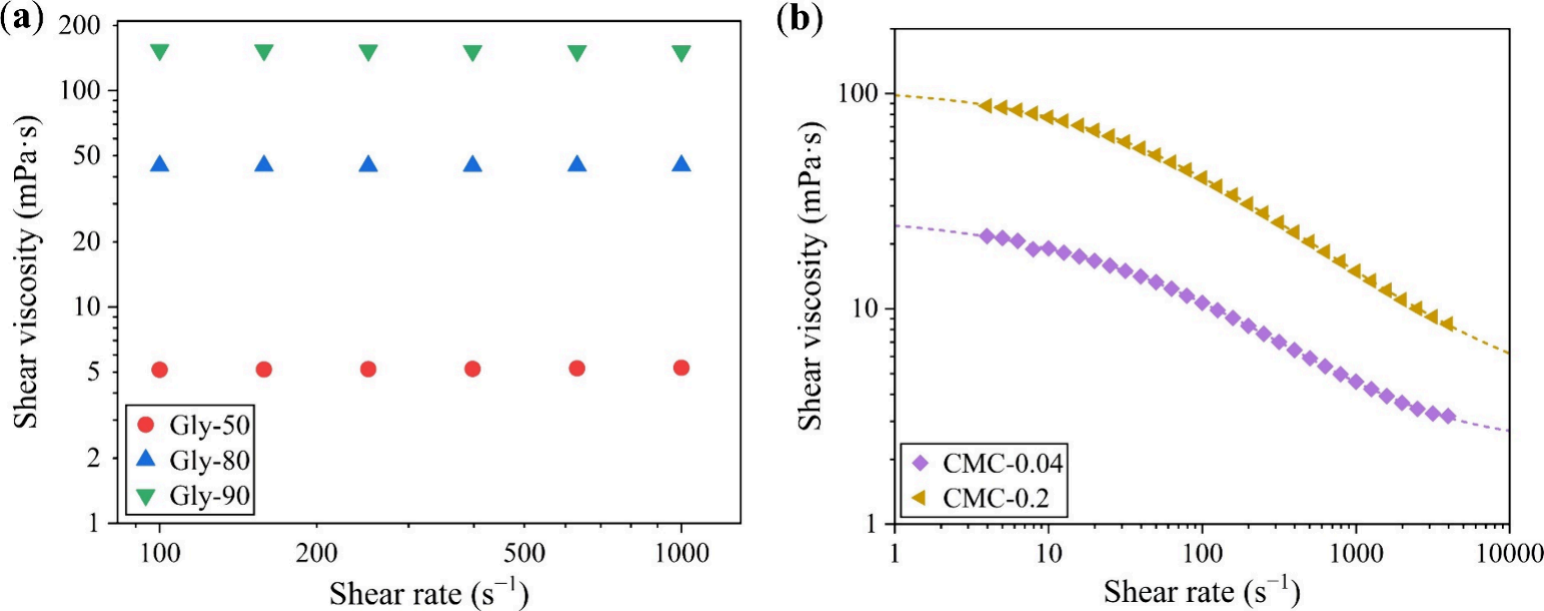
Shear viscosity of the prepared liquid samples. Shear viscosity plotted as a function of shear rate for (a) glycerol–water mixtures and (b) CMC solutions. Shear viscosity measurements were conducted on a rotational rheometer. The dashed lines in panel b are the fitted curves obtained using the Carreau–Yasuda viscosity model.

Figure 1b illustrates the shear viscosity of CMC solutions at two different concentrations as a function of shear rate, both featuring a pronounced shear-thinning profile. The higher-concentration solution (CMC-0.2) consistently exhibits greater viscosity than the lower one (CMC-0.04). The data were fitted using the Carreau–Yasuda viscosity model,^37^ with the results depicted as dashed lines in Figure 1b. The fitted curves showcase a good agreement with the experimental data, enabling precise determination of shear viscosity at any given shear rate. Consequently, we prepared non-Newtonian fluids with shear-thinning properties at different viscosity levels for subsequent droplet impact experiments.

### Morphological Observations of Droplet Impact

We conducted droplet impact experiments using the prepared Newtonian and shear-thinning (non-Newtonian) fluids to investigate the relationship between droplets’ viscosity and their spreading dynamics. To minimize the influence of surface wettability on droplet spreading, a super-repellent surface was employed as the solid substrate for impact and subsequent spreading. Figure 2a presents a schematic diagram of the experimental setup for droplet impact. Upon release from the needle tip, the droplet falls vertically and impacts the underlying super-repellent surface, with a high-speed camera capturing the whole process. The impact velocity *U* can be calculated using information from the high-speed videos. Snapshots of the impact and spreading processes are shown in Figure 2b, featuring Water as a representative Newtonian fluid and CMC-0.2 as a representative shear-thinning fluid. Both liquids exhibit similar shape variation and spreading behavior: upon contacting the surface, a droplet with an initial diameter *D*_0_ spreads horizontally until it reaches the maximum spreading, characterized by a maximum diameter *D*_m_. When the impact inertia is sufficiently large, the droplet becomes remarkably flattened at the maximum spreading, resembling a short cylinder. Subsequently, the droplet recedes and eventually detaches from the surface without leaving any residue, owing to the liquid-repellent property of the superamphiphobic substrate.^38^

**Figure 2:**
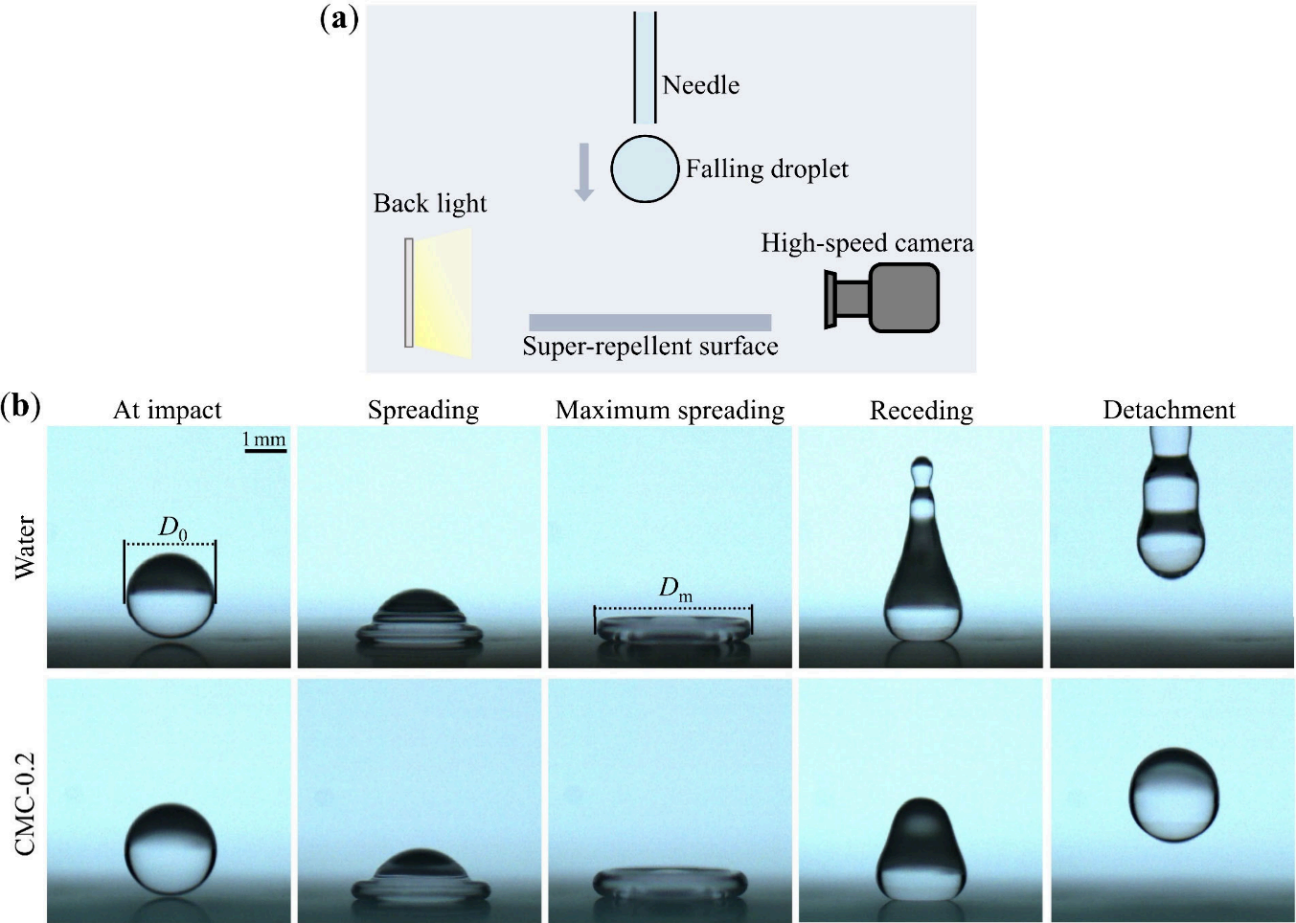
Illustration of droplet impact. (a) Schematic diagram of the experimental setup for droplet impact. A droplet released from the needle falls and impacts the super-repellent surface, with the impact event recorded by a high-speed camera. (b) Snapshots showing droplets at various stages: at impact, spreading, maximum spreading, receding, and detachment. Water represents a Newtonian fluid and CMC-0.2 represents a shear-thinning fluid (non-Newtonian). The impact velocities of the droplets for Water and CMC-0.2 are 0.78 m s^–1^ and 0.77 m s^–1^, respectively.

As the inertial force increases with the gradual rise in impact velocity *U*, small fingerings begin to emerge along the edge of droplets at the maximum spreading due to rim instability (see the middle column in Figure 3).^39^ A further increase in *U* exacerbates the fingering patterns, and can even lead to the splashing of water droplet, because of its low viscosity (see the right column in Figure 3). Notably, for the highest-viscosity liquid in this study, Gly-90, a different structure was observed: a thin liquid sheet ejected from a slightly thicker central layer at high impact velocities, potentially representing a precursor to splashing in viscous liquids.^40^ To ensure accurate and robust measurement of the maximum spreading factor β, we restricted the range of impact velocity in our study to avoid the interference of fingering and splashing effects.

**Figure 3:**
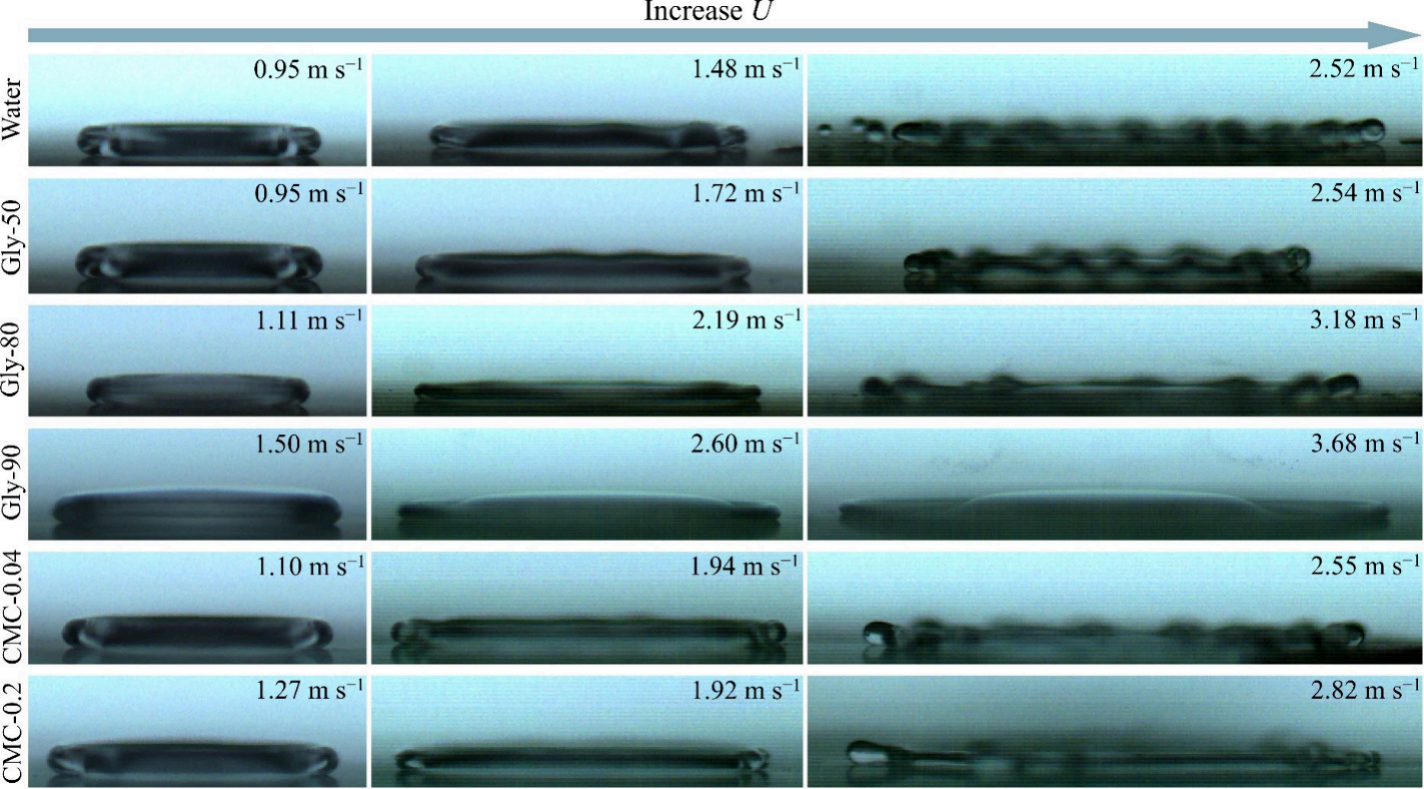
Snapshots of droplet shapes at the maximum spreading for different impact velocities. The impact velocity *U* increases progressively from the left column to the right column.

### Comparison of Experimental and Predictive β Values

Many efforts have been devoted to understanding the spreading dynamics of droplet impact, and numerous plausible yet distinct scaling laws and models have been proposed for predicting the maximum spreading factor β. In this subsection, we examined the compatibility of the experimental β values for the Newtonian droplets in our study against several widely referenced scaling laws and models from the literature.

We began by analyzing the relationship between β and two dimensionless numbers, the Weber number (We) and the Reynolds number (Re), which are utilized to compare inertia with capillary and viscous forces, respectively. The Weber number is defined as We = *ρU* ^2^ *D*_0_/σ, and the Reynolds number as Re = *ρUD*_0_/η, where ρ is the density, *U* the impact velocity, *D*_0_ the initial diameter, σ the surface tension, and η the shear viscosity measured by the rheometer, respectively. Typically, two opposing regimes—capillary and viscous regimes—are distinguished to explain the spreading behavior of droplets.^31^ In the capillary regime, viscous forces are negligible, so the interplay between inertia and capillarity is employed for scaling analyses, resulting in two commonly cited relationships: β ∼ We^1/4^, deduced using the effective capillary length based on momentum and mass conservation,^41^ and β ∼ We^1/2^, obtained from an energy conservation argument.^42^ Conversely, in the viscous regime, where viscous forces dominate over capillarity, the scaling β ∼ Re^1/5^ is derived by balancing kinetic energy with viscous dissipation.^28^ However, as depicted in Figure 4, the data from our impact experiments do not collapse onto a single line, indicating that none of the aforementioned simple scaling laws (represented by dashed lines) can universally describe the spreading behavior. In our cases, β may result from a complex interplay among inertial, capillary, and viscous forces. Consequently, the spreading dynamics cannot be adequately characterized within the constraints of any single limiting regime, whether capillary or viscous.

**Figure 4:**
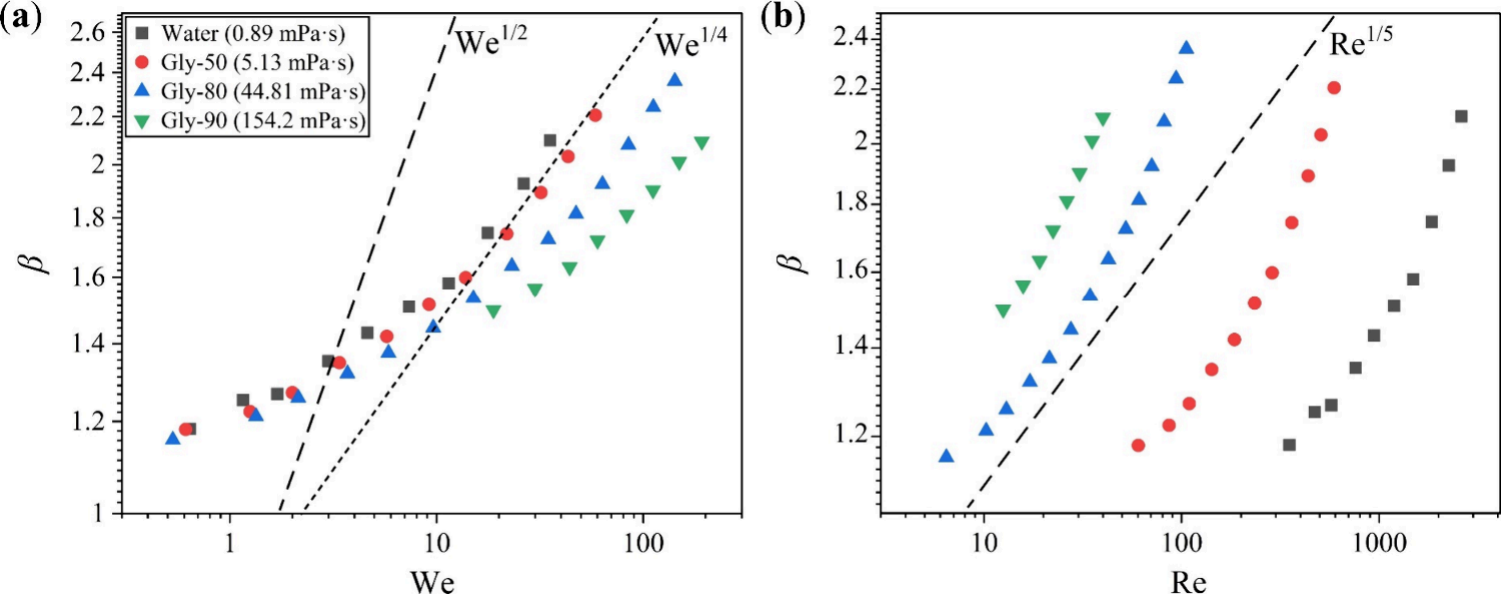
Maximum spreading factor β of the Newtonian fluids as a function of (a) the Weber number (We) and (b) the Reynolds number (Re). The dashed lines represent the scaling laws β ∼ We^1/2^ and β ∼ We^1/4^ in panel a and β ∼ Re^1/5^ in panel b.

To explain the spreading dynamics with intricate balance between various effects, interpolation between different scaling relations has been introduced. Laan et al.^31^ proposed a rescaling of β between We^1/2^ and Re^1/5^, resulting in a master curve of the form:

1 βRe−1/5=We1/2Re−1/5A1+We1/2Re−1/5

where *A*_1_ is a fitting constant. This equation enables the prediction of β based on We and Re numbers. However, we found that the predicted values, β_model_, calculated using the model of eq 1, significantly diverged from our experimental values, β, as shown in Figure 5a. The relative mean error is 2.70% with a standard deviation of 22.9% (Table 2), indicating a poor accuracy in predicting β for our data set. This discrepancy can be attributed to the rescaling curve’s failing to unify the data (see Figure S1a), particularly at low impact velocities. Even when the data set was restricted to points with *U* > 0.9 m s^–1^ to align with the impact velocity condition employed in the experiments of Laan et al., the fitted curve still remarkably deviated from the data points (Figure S1b).

**Figure 5:**
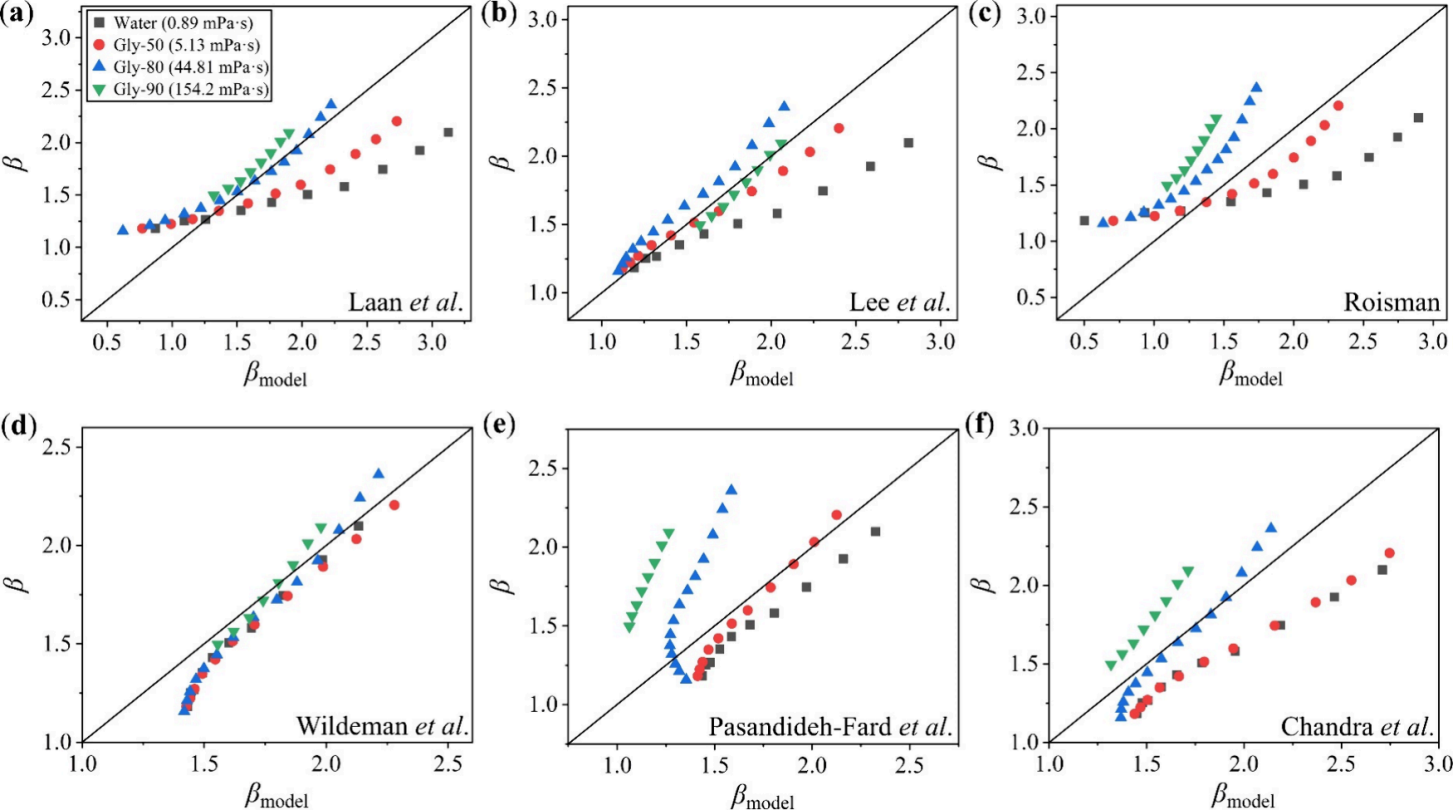
Experimental β values of the Newtonian fluids in this study plotted against predictive values β_model_ obtained from models by (a) Laan et al.,^31^ (b) Lee et al.,^32^ (c) Roisman,^43^ (d) Wildeman et al.,^35^ (e) Pasandideh-Fard et al.,^44^ and (f) Chandra et al.^45^ The black lines indicate the ideal relationship β = β_model_.

**Table 2:** Relative Mean Error and Corresponding Standard Deviation of *β*_model_ Predicted by Different Models

Model	Relative mean error (%)	Standard deviation (%)
Laan et al.^31^	2.70	22.9
Lee et al.^32^	2.25	12.1
Roisman^43^	–8.29	25.5
Wildeman et al.^35^	6.69	7.09
Pasandideh-Fard et al.^44^	–5.96	19.9
Chandra et al.^45^	8.71	14.7
This work (Newtonian fluids)	–0.086	1.38
This work (shear-thinning fluids)	–0.737	1.22

Similarly, based on the interpolation method, Lee et al.^32^ proposed a corrected scaling relation that is applicable to a wider range of impact velocity by taking into account the capillary energy due to wetting in the limit of zero impact velocity (*U* → 0 m s^–1^):

2 (β2−β02)1/2Re−1/5=We1/2A2+We1/2

where *A*_2_ is a fitting constant, and β_0_ represents the maximum spreading factor as *U* approaches zero, approximated through an empirical function. We applied Lee et al.’s model to evaluate our data; however, it yielded a poor fit, as displayed in Figure 5b, with a relative mean error of 2.25% ± 12.1%. The scaling curve also failed to collapse our data points (Figure S2).

Roisman^43^ developed a semiempirical scaling relation by employing a dynamical model that involves a viscous boundary layer, expressed as:

3 β=A3Re1/5−B3Re2/5We−1/2

where *A*_3_ and *B*_3_ are fitting constants. However, this model cannot accurately represent our data, as the predicted β_model_ significantly diverged from the experimental β (Figure 5c), with an error of – 8.29% ± 25.5%.

Through numerical simulations of droplet impact, Wildeman et al.^35^ identified an empirical 1/2-rule that suggests approximately half of the droplet’s initial kinetic energy is converted into viscous dissipation under free-slip conditions. For no-slip conditions, they applied this 1/2-rule while accounting for viscous dissipation within the boundary layer, deriving an equation for β based on energy conservation among kinetic, surface, and viscous dissipation energies:

4 3(1−cos⁡θ)Weβ2+A4Reβ2β−1=12We+12

where θ is the contact angle and *A*_4_ is a fitting constant, respectively. β_model_ is obtained by solving eq 4 numerically. Nevertheless, as shown in Figure 5d, the model does not accurately describe our data, showing an error of 6.69% ± 7.09%. Even when restricting We > 30 to match the impact condition required in the study of Wildeman et al., achieving precise predictions remains challenging (Figure S3).

Two additional classical models based on detailed energy balance were also utilized to compare with our experimental data. The model reported by Pasandideh-Fard et al.^44^ is expressed as:

5 β=We+123(1−cos⁡θa)+4WeRe

where *θ*_*a*_ is the advancing contact angle. The model introduced by Chandra et al.^45^ is given by:

6 32WeReβ4+(1−cos⁡θ)β2−(13We+4)=0

After calculating the predictive β_model_, we found that both models exhibited poor predictive performance for our experimental data (see Figure 5e,f). The model by Pasandideh-Fard et al. yielded an error of – 5.96% ± 19.9%, while the model by Chandra et al. resulted in an error of 8.71% ± 14.7%.

The relative mean errors and standard deviations of all models examined using our data are summarized in Table 2. The large errors and standard deviations indicate these reported models are not universally applicable for describing the spreading dynamics of liquids with the viscosity range investigated in our experiments, suggesting their limitations.

### A Semiempirical Model for Predicting β

To better describe the spreading behavior, we conducted a scaling analysis by balancing the reinforced gravity effect with capillary and viscous effects. At the maximum spreading, the droplet gets flattened remarkably into a short, cylinder-like shape. The instantaneous deformation of droplet induced by impact can be equivalently interpreted as resulting from a reinforced gravity^41^ that deforms the droplet by overcoming capillary and viscous forces, whereby a balance is established as *ρah* ∼ (σ/*h* + *ηU*/δ), where *a* is the effective gravitation acceleration and δ represents the thickness of the viscous boundary layer. Assuming the droplet’s velocity decreases from *U* to 0 over a spreading time *t*_m_ of the order of *D*_m_/*U*, the acceleration scales as *U* ^2^/*D*_m_. The viscous boundary layer thickness at the maximum spreading is approximated by δ ∼ $\sqrt{\eta t_{m}/\rho }$ .^35^ Further using the volume conservation relationship *hD*_m_^2^ ∼ *D*_0_^3^, we deduce a maximum spreading factor:

7 β∼(We−1+β−5/2Re−1/2)−1/5

which suggests that β scales as the 1/5 power of (We^–1^ + β^–5/2^Re^–1/2^)^−1^. As shown in Figure 6a, this scaling law matches our experimental data well within the range β = 1.50 to 1.95, where the volume conservation relationship is most valid due to the droplet geometry at maximum spreading closely resembling a short cylinder.^46^ Moreover, this scaling successfully collapses the data for the Newtonian fluids with varying viscosities onto a single master curve, represented by the black dashed line in Figure 6a. Assuming β approaches 1 in the limit of zero impact velocity, the equation for the master curve is derived by fitting the experimental data using the following formula:

8 $$\beta ={\left(Ax^{B}+1\right)}^{C}$$

where *x* = (We^–1^ + β^–5/2^Re^–1/2^)^−1^ represents the horizontal axis in Figure 6a, and *A* = 0.0698, *B* = 0.403, *C* = 2.89, are fitting constants. The fit yields a coefficient of determination *R* ^2^ of 0.997, showcasing a good agreement between the data and fitted curve. Therefore, we obtained a semiempirical formula that is capable of predicting β from the impact numbers by solving eq 8 numerically. As shown in Figure 6b, the predicted values β_model_ align closely with experimental values β, achieving an error of – 0.086% ± 1.38% (Table 2). This result demonstrates the formula’s good applicability for Newtonian fluids across different viscosity ranges.

**Figure 6:**
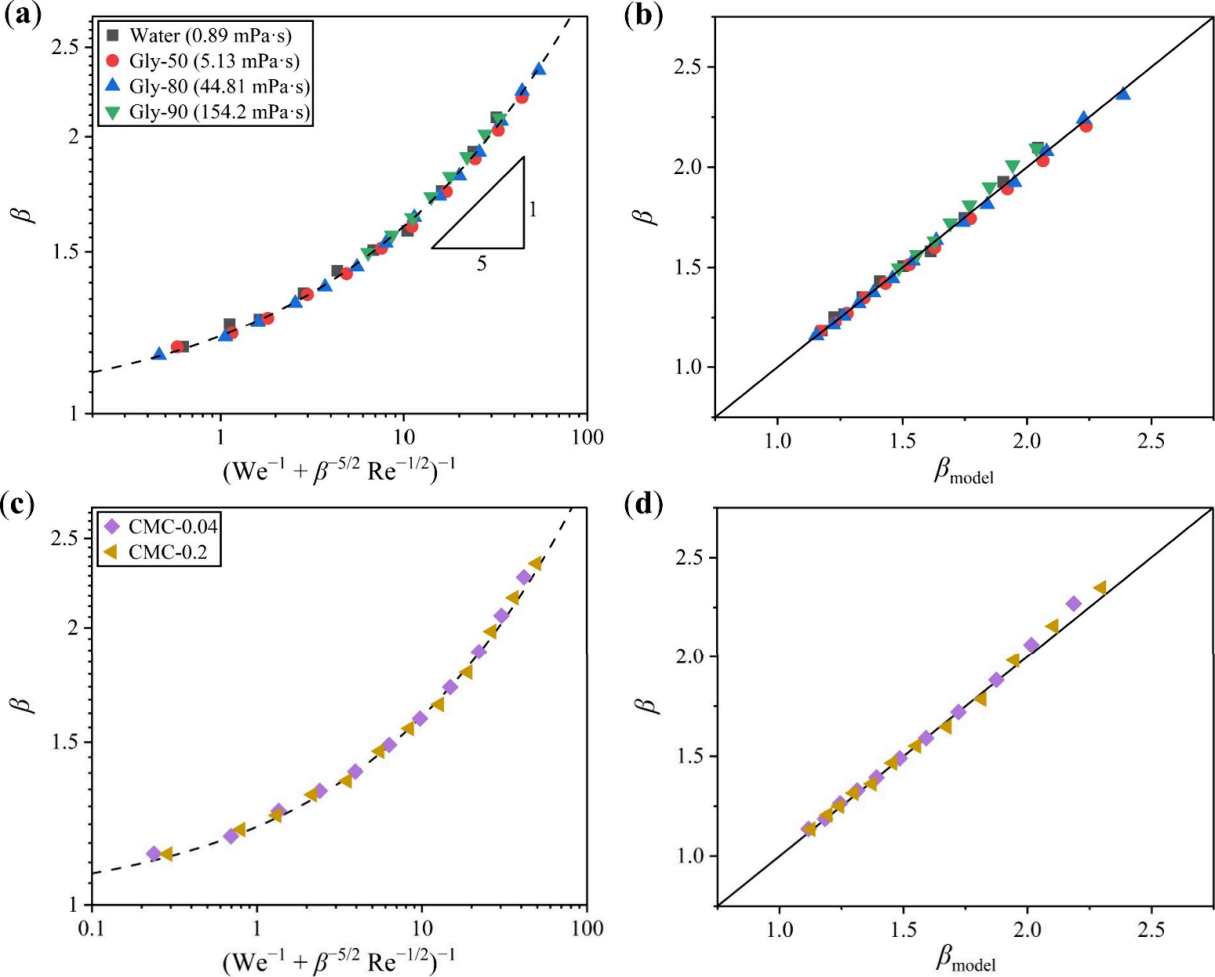
β as a function of (We^–1^ + β^–5/2^Re^–1/2^)^−1^ for (a) the Newtonian fluids and (c) the shear-thinning (non-Newtonian) fluids, with the dashed lines representing eq 8. Experimental β values versus predictive values β_model_ obtained by solving eq 8 are shown for (b) the Newtonian fluids and (d) the shear-thinning (non-Newtonian) fluids, with the straight lines indicating the ideal relationship β = β_model_.

We further examined whether this semiempirical model applies to shear-thinning (non-Newtonian) fluids. To calculate the Reynolds number for the shear-thinning CMC solutions in our study, their shear viscosity was obtained from the fitted curves in Figure 1b. The effective shear rate *γ̇* was estimated by the velocity gradient, *γ̇* = *U*/*h*, with the height *h* approximated as *h* = 2*D*_0_^3^/3*D*_m_^2^ based on volume conservation.^45,47^ The results, displayed in Figure 6c, demonstrate that eq 8 also effectively collapses the data for the two shear-thinning fluids with different viscosity levels. The precision of the predictions is further highlighted in Figure 6d, which has an error of – 0.737% ± 1.22% in predicting β. It should be noted that the semiempirical formula used to calculate the β_model_ for both the Newtonian and shear-thinning fluids is identical. Interestingly, the fitting constants *A*, *B*, and *C* remain consistent and unchanged, although they were obtained from fitting the data of Newtonian fluids.

These findings demonstrate that the semiempirical model we developed in this study is applicable for predicting the maximum spreading factor β for both the Newtonian and the shear-thinning (non-Newtonian) fluids. Moreover, our model exhibits a higher accuracy compared with several classical models reported in the literature (Table 2).

### Viscosity Measurement and Error Analysis

The good accuracy of the semiempirical model we proposed suggests the potential of using β as a metric to characterize the viscosity properties of liquids. From eq 8, we can derive an analytical expression for calculating the viscosity, denoted as η_c_:

9 $$\eta_{c}=\rho UD_{0}\beta^{5}{\left[{\left(\frac{A}{\beta^{1/C}-1}\right)}^{1/B}-{\mathrm{We}}^{-1}\right]}^{2}$$

The droplet impact experiments were conducted at varying impact velocities, effectively corresponding to different shear rates experienced by the droplets. Thus, from each droplet impact test at a specific impact velocity, we can obtain a corresponding viscosity. The viscosities η_c_ calculated from eq 9 are compared with standard viscosity values η obtained from the rheometer. Interestingly, the agreement between η_c_ and η appears to be influenced by the viscosity of the liquids and the applied shear rate.

We subsequently analyzed the sources of discrepancies between η_c_ and η. The relative error between η_c_ and η is plotted as a function of a dimensionless ratio *r* (Figure 7), defined as *r* = β^–5/2^Re^–1/2^/We^–1^, which is the ratio of the two additive terms in eq 7. By introducing the Capillary number Ca = *ηU*/σ, *r* can be expressed as *r* = We^1/2^Ca^1/2^/β^5/2^, reflecting the relative magnitude of the inertia and the viscous forces compared with the surface tension. As shown in Figure 7, when *r* < 0.1, the viscous force is minimal compared to the capillary force, thereby exerting a negligible influence on β. As a result, the term ${\left(\frac{A}{\beta^{1/C}-1}\right)}^{1/B}-{\mathrm{We}}^{-1}$ in eq 9, which represents the viscous component after subtracting the capillary component, is exceedingly small so that even minor experimental errors in measurement can significantly influence it, leading to large deviations between η_c_ and η. Since experimental errors are random, the relative error in viscosity exhibits random fluctuations, as exemplified by the data for Water in Figure 7. In contrast, when *r* > 0.1, the inertia and the viscous forces are comparable to the capillary force. As such, the viscous effect becomes sufficient, and the subtracting term in eq 9, ${\left(\frac{A}{\beta^{1/C}-1}\right)}^{1/B}-{\mathrm{We}}^{-1}$ , is of appreciable magnitude, making η_c_ less sensitive to experimental errors.

**Figure 7:**
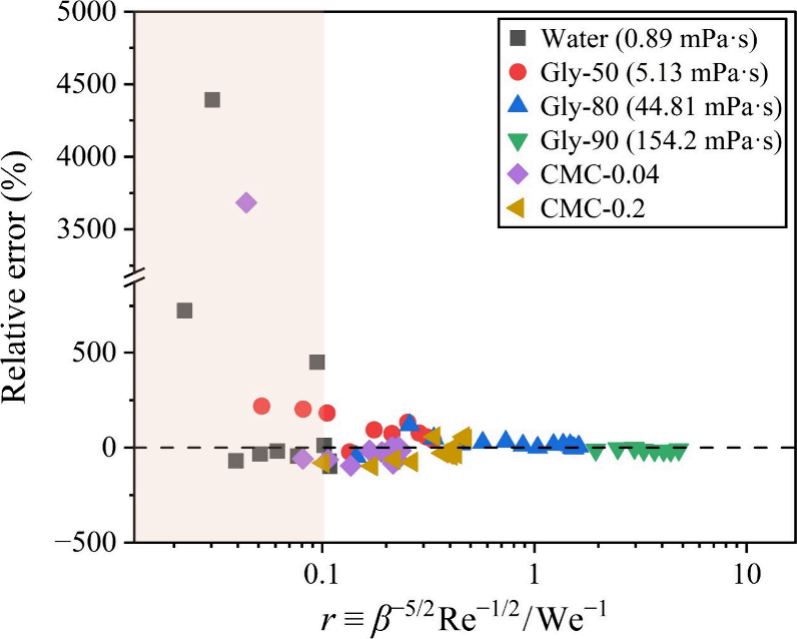
Relative error between the calculated viscosity η_c_ using eq 9 and the standard viscosity η from the rheometer plotted as a function of the dimensionless ratio *r* = β^–5/2^Re^–1/2^/We^–1^. The dashed line indicates the ideal case of zero error.

### Droplet Impact-Based Microliter Viscometry

The results presented above suggest that the sufficient viscous effect during droplet impact can be utilized to measure liquid viscosity. Specifically, for a liquid sample with unknown viscosity, we can calculate a viscosity η_c_ using eq 9 based on the droplet impact data. To assess the reliability of η_c_, analogous to *r*, we define a dimensionless ratio *r*_c_ = β^–5/2^Re_c_^–1/2^/We^–1^, where Re_c_ = *ρUD*_0_/η_c_ is the Reynolds number calculated using the viscosity η_c_. If *r*_c_ > 0.1, the viscous effect is sufficient, and the calculated viscosity η_c_ is considered reliable; otherwise, the obtained η_c_ is deemed unreliable. We designate this method as droplet impact-based microliter viscometry (DI-μV) in this study. It is worth noting that, due to the coexistence of shear and extensional flows during droplet impact, the viscosity measured using the DI-μV method should be regarded as an effective viscosity. Furthermore, due to the difficulty in resolving the spatially and temporally varying shear rates during the impact process, we make an estimation by taking the ratio of the initial impact velocity to the droplet height as an effective shear rate.

The effective viscosities obtained through DI-μV method are presented as scatters in Figure 8. For fluids without significant extensional viscosities, the relationship between effective viscosity and effective shear rate can be utilized to analyze the shear dependency of a fluid, enabling the qualitative determination of whether it is Newtonian or non-Newtonian. For this purpose, we applied a power-law viscosity model, η = *Kγ̇* ^n–1^, where *K* is the flow consistency index, and *n* is the flow behavior index, to fit the effective viscosities obtained from DI-μV. The value of *n* in the power-law model determines the fluid type: *n* = 1 indicates a Newtonian fluid, while *n* < 1 corresponds to a shear-thinning (non-Newtonian) fluid. The fitted *n* values and corresponding curves are presented in Figure 8. For the glycerol–water mixtures, the fitted *n* values are very close to 1, indicating minimal dependency of effective viscosity on effective shear rate, thereby classifying them as Newtonian fluids. In contrast, for CMC solutions, the fitted *n* values are significantly lower than 1, confirming that their effective viscosity decreases with increasing effective shear rate, identifying them as shear-thinning (non-Newtonian) fluids.

**Figure 8:**
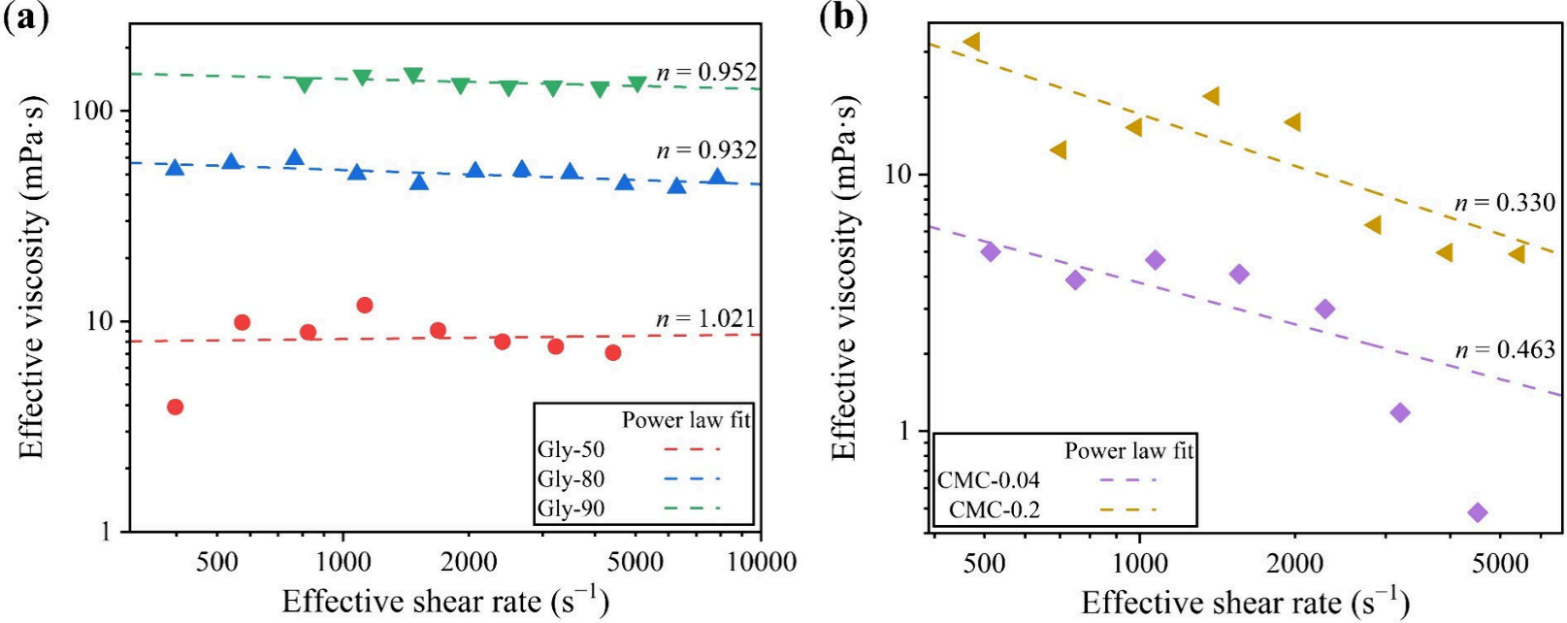
Effective viscosity plotted as a function of effective shear rate for (a) the Newtonian fluids and (b) the shear-thinning (non-Newtonian) fluids, with the symbols representing viscosity values obtained from DI-μV method and the dashed lines indicating the fitted results by using the power-law viscosity model.

For Newtonian fluids, the average value of effective viscosities obtained from DI-μV method across different effective shear rates can be calculated to mitigate the influence of experimental errors. In Figure 9, these averaged values are compared with the standard shear viscosity values measured using the rheometer. For Gly-50, which has a relatively low viscosity, the corresponding *r*_c_ values (0.12–0.40) are not significantly greater than 0.1. Consequently, the averaged effective viscosity obtained via DI-μV for Gly-50 exhibits minor deviation from the standard shear viscosity. However, as the fluid viscosity increases, the *r*_c_ values for Gly-80 (0.49–1.67) and Gly-90 (1.83–4.52) are much greater than 0.1, leading to effective viscosity values that are consistent with the shear viscosities obtained from the rheometer. These findings suggest that DI-μV method achieves a good accuracy in effective viscosity measurement when the viscous effect during droplet impact is sufficient, as characterized by the relatively high *r*_c_ values. It is important to note that, since the effective shear rate is not numerically equivalent to the well-defined shear rate measured in conventional rheometers, our method is more appropriate for characterizing the effective viscosity of Newtonian fluids and weakly non-Newtonian fluids, such as low-molecular-weight polymer solutions.

**Figure 9:**
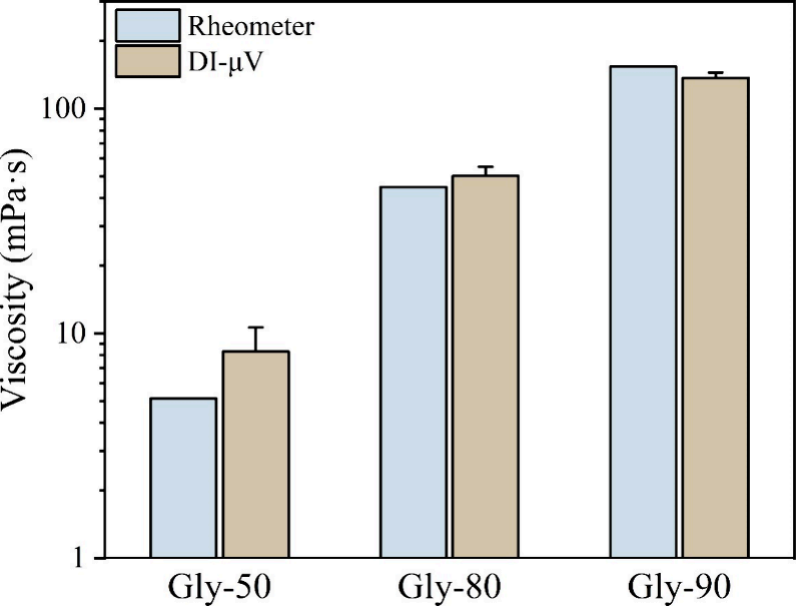
Comparison of viscosity measurements for the Newtonian fluids obtained by different methods: average effective viscosity values obtained from DI-μV method versus standard shear viscosity values from the rheometer.

Nevertheless, DI-μV method remains useful for characterizing the viscosity properties of small-volume liquids due to its minimal sample consumption. In our current setup, each droplet has a diameter of approximately 2 mm, corresponding to a volume of about 4.2 μL. Since each impact yields a single measurement of the effective viscosity, triplicate measurements at a given effective shear rate require only 12.6 μL of liquid. To investigate the viscosity behavior across a range of effective shear rates, the total sample volume required is proportional to the number of discrete sampling points of effective shear rate; for example, approximately 126 μL of sample is needed for ten different effective shear rates. Thus, the typical sample volume required by DI-μV ranges from ∼ 10 μL to ∼ 100 μL (for multiple effective shear rate conditions), which is significantly less than the milliliter-scale volumes demanded by conventional viscometers or rheometers.

## Conclusions

In summary, this study presents a semiempirical predictive model for determining the maximum spreading of droplets impacting super-repellent surfaces. Our model shows a good agreement with experimental results for both Newtonian and shear-thinning (non-Newtonian) fluids. Based on our model, a quantitative relationship between viscosity and β is established. The sources of error in viscosity calculation are thoroughly analyzed, determining the criterion to evaluate the reliability of calculated viscosity values. Furthermore, we introduce the droplet impact-based microliter viscometry (DI-μV), which is demonstrated to effectively measure the effective viscosity at different effective shear rates and probe the rheological properties of liquids, using only μL-scale sample volumes. In addition, the substrate used for droplet impact in DI-μV is a self-cleaning super-repellent surface, which prevents the contamination of equipment and simplifies cleaning processes.^48^ We envision that DI-μV integrates several distinctive advantages, including low sample consumption, adjustable effective shear rate, self-cleaning capabilities, operational simplicity, and cost-effectiveness. These attributes position DI-μV as a promising measurement tool for applications in fields such as biomedical diagnostics and chemical product analysis.

## Supplementary Material



## Notes

Fitted curves of eq 1 (Figure S1) and eq 2 (Figure S2), comparison between experimental β values and predictive values β_model_ obtained using the model of eq 4 under the condition of We > 30 (Figure S3) (PDF)
