# Supplementary material for: Droplet Impact-Based Microliter Viscometry
Source: Anal Chem. 2025 Jun 16;97(25):13076–85. doi: 10.1021/acs.analchem.5c00471 (PMC12713610; doi:10.1021/acs.analchem.5c00471)
Supplement: Supplementary file 1 [file ac5c00471_si_001.pdf]

Supporting Information for

# Droplet impact-based microliter viscometry

Shuxian Tang,[1] Xiang Li,[2] Wanying Wang,[3] Wenchang Zhao,[1] Ying Zhou,[1] Shiyu Wang,[1] Yanhong Li,[1] Peng Yu,[2] Xiewen Wen,[4] Guohui Hu,[5] and Pingan Zhu[1,*]

[1]Department of Mechanical Engineering, City University of Hong Kong, 999077 Hong Kong, China

[2]Department of Mechanics and Aerospace Engineering, Southern University of Science and Technology, 518055 Shenzhen, China

[3]Department of Biomedical Sciences, City University of Hong Kong, 999077 Hong Kong, China

[4]State Key Laboratory of Ultra-precision Machining Technology, Department of Industrial and Systems Engineering, The Hong Kong Polytechnic University, 999077 Hong Kong, China

[5]Shanghai Institute of Applied Mathematics and Mechanics, School of Mechanics and Engineering Science, Shanghai Key Laboratory of Mechanics in Energy Engineering, Shanghai Frontier Science Center of Mechanoinformatics, Shanghai University, 200072 Shanghai, China

*Author to whom correspondence should be addressed: pingazhu@cityu.edu.hk.

**Table of contents:**

**Figure S1.** $\beta\mathrm{Re}^{-1/5}$ plotted as a function of $\mathrm{WeRe}^{-2/5}$.

**Figure S2.** $(\beta^2 - \beta_0^2)^{1/2}\mathrm{Re}^{-1/5}$ plotted as a function of We.

**Figure S3.** Experimental $\beta$ values of the Newtonian fluids (We > 30) plotted against predictive values $\beta_{\mathrm{model}}$ obtained from the model by Wildeman *et al*.

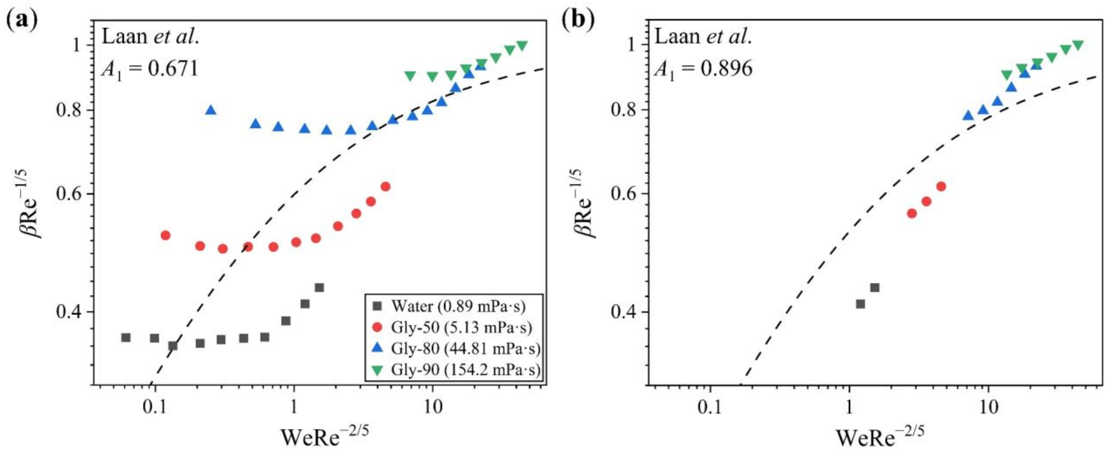


**Figure S1.** $\beta\mathrm{Re}^{-1/5}$ plotted as a function of $\mathrm{WeRe}^{-2/5}$ for (a) all data points, and (b) data points with impact velocity $U > 0.9$ m s$^{-1}$. The dashed lines represent fits to the data using Eq. (1) from the model by Laan *et al*.

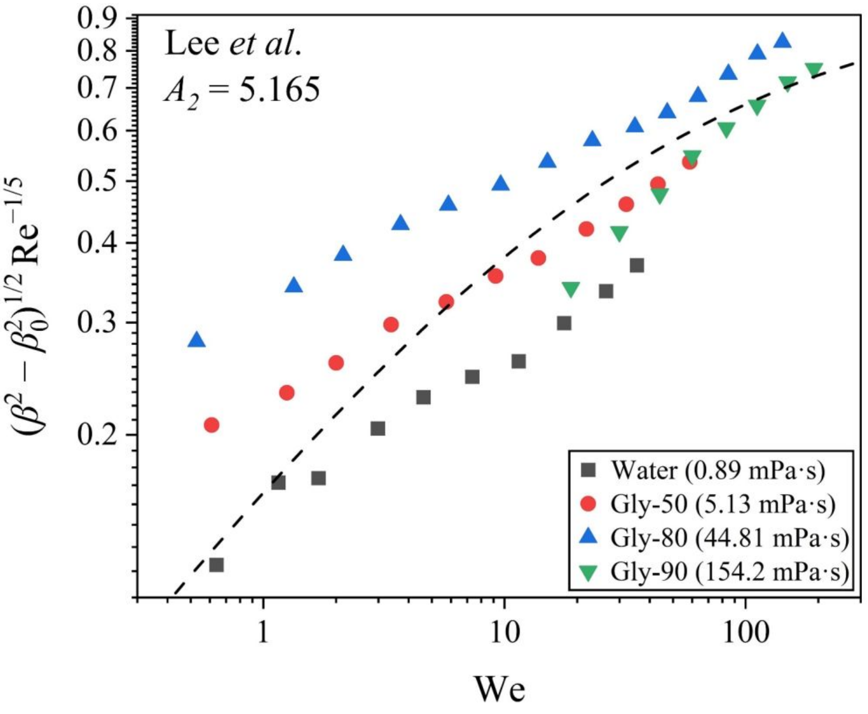


**Figure S2.** $(\beta^2 - \beta_0^2)^{1/2}\mathrm{Re}^{-1/5}$ plotted as a function of We. The dashed line represents the fit to the data using Eq. (2) from the model by Lee *et al*.

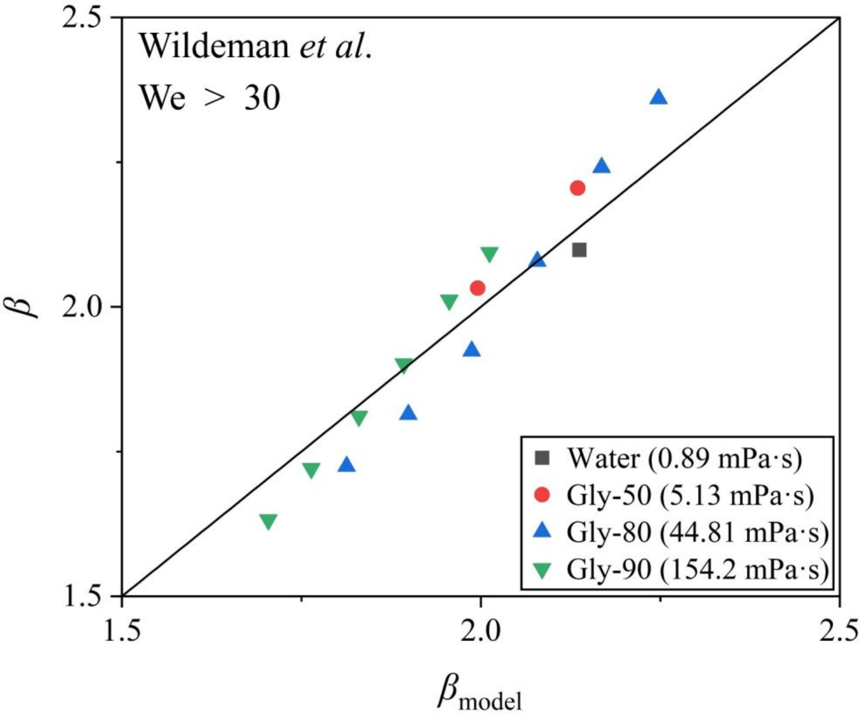


**Figure S3.** Experimental $\beta$ values of the Newtonian fluids (We > 30) plotted against predictive values $\beta_{\mathrm{model}}$ obtained from the model by Wildeman *et al*. The black line indicates the ideal relationship $\beta = \beta_{\mathrm{model}}$.
